# Student-athletes at risk: faculty voices on gender-based violence and the need for structural change in physical activity and sport sciences programs

**DOI:** 10.3389/fspor.2026.1736683

**Published:** 2026-02-26

**Authors:** Pedrona Serra Payeras, Susanna Soler-Prat, Carlos Matus-Castillo, Ingrid Hinojosa-Alcalde

**Affiliations:** 1 Departamento de Pedagogía y Didácticas Específicas, University of the Balearic Islands, Grup d'Investigació en Ciències de l'Activitat Física i l'Esport (GICAFE), Palma, Spain; 2 Institut Nacional d'Educació Física de Catalunya (INEFC), Universitat de Barcelona (UB), Grup d'Investigació Social Educatiu de l'Activitat Física i l'Esport (GISEAFE), Barcelona, Spain; 3 Facultad de Educación, Universidad Católica de la Santísima Concepción, Concepción, Chile

**Keywords:** code of conduct, faculty perceptions, gender equality policies, gender-based violence, higher education, physical activity and sport sciences, sport protection

## Abstract

Creating Gender-Based Violence (GBV) free environments remains a significant challenge in the fields of physical activity, sport, and higher education. This study examines the perceptions, experiences, and proposals of university teaching staff regarding GBV within a Physical Activity and Sport Sciences (PASS) programs. Three focus groups were conducted with 17 faculty members (eight women and nine men). Data was analyzed through a reflexive thematic analysis complemented by 7P model as an analytical framework. Findings show that while teaching staff can identify overt forms of violence such as sexual and physical abuse, and recognize the existence of structural and organizational inequalities within PASS programs. However, participants report significant uncertainty regarding how to intervene in GBV cases involving students, limited knowledge of institutional protocols, and insufficient procedural guidance. Across the dimensions of Policy, Prevention, Protection, Prosecution, Provision, Prevalence, and Partnerships, the results reveal uneven institutional development, with particular weaknesses in prevention strategies, dissemination of policies, reporting processes, and victim support mechanisms. Faculty discourses also highlight tensions between victim protection and the prioritization of alleged perpetrators’ rights. The study underscores the urgent need for comprehensive training programs for academic staff and students, clearer governance structures, and coordinated support systems to address GBV in sport-related higher education. By applying the 7P framework to the PASS context, this research contributes empirical evidence to inform more coherent, accountable, and gender-responsive institutional policies and practices.

## 1 Introduction

Gender-Based Violence (GBV), defined as violence exercised against a person because of their gender or that which disproportionately affects people of a specific gender (1) remains a persistent issue in spaces linked to physical activity, physical education, and sport (2–4). The power relations underpinning these forms of violence and the gendered culture of sport have often shaped not only sporting practices but also the institutional cultures of higher education, particularly within sport sciences and physical education teacher education. Such environments may reproduce hierarchies and exclusions that marginalize those who do not conform to gender norms (5). Within this context, female and non-normative students are exposed to multiple forms of gender-based violence, including sexual harassment, objectification, media hypersexualization, and discrimination based on gender or sexual orientation (6, 7). Furthermore, teachers’ lack of training in GBV and gender issues leaves them unable to recognize or act on these situations, thereby reinforcing impunity and perpetuating these dynamics (8–11). This lack of preparation represents an urgent challenge for teacher training and institutional policies in the fields of higher education and sports. While knowledge of GBV is necessary but not sufficient for a person (teacher, student, coach) to intervene in a situation of GBV, it is also necessary to challenge harmful social norms, promote favorable attitudes, and improve self-confidence in the ability to act (12).

GBV is a global problem with high prevalence in sports and university settings (13). It represents one of the most persistent expressions of inequality in contemporary societies (14, 15). It is not merely an isolated phenomenon, but a structural and sustained form of discrimination against women, as noted by the Office of the United Nations High Commissioner for Human Rights (16). Its impact transcends any specific sphere and manifests itself in multiple social spaces, including those that have traditionally been considered neutral or protective, such as higher education (17) and sport (18, 19). To adequately address GBV, it is important to recognize the root causes of violence, specifically the gendered nature of violence embedded in unequal gendered power structures, gender inequality, and discrimination. This requires a more comprehensive approach that includes prevention measures and efforts to promote gender equality, and a conceptual shift from violence against women to gender-based violence (20).

University programs in Physical Activity and Sport Sciences (PASS) are critical settings where future sport professionals are trained, and therefore represent key spaces for either reinforcing or challenging gender inequalities. Understanding how faculty perceive GBV, institutional responses, and existing equality protocols is essential for informing gender-sensitive sport policies and strengthening prevention and governance structures in higher education.

GBV is not limited to explicit or visible harm; it is often expressed in subtle, covert, or symbolic ways, reproduced through discourse, everyday practices, or institutional silence (21, 22). Sexual harassment, physical assault, discrimination, exclusion, and ridicule are examples of how these dynamics intersect with other systems of oppression such as class, race, and sexual orientation, creating unequal experiences for those who inhabit academic and sporting spaces (23). This intersection of inequalities confirms that the analysis of GBV cannot be limited to simple or isolated categories.

Traditionally, a distinction has been made between physical, sexual, psychological, economic, and symbolic forms (24), but specific types such as institutional, structural, and digital have also been identified (25, 26). It is important to note that these categories often overlap and reinforce each other. The omission of protocols for dealing with cases of harassment or the lack of sanctions for abusive behavior, for example, are expressions of institutional violence that contribute to the reproduction of direct or symbolic violence (27). Thus, rather than a descriptive exercise, this classification offers a way to understand the complexity and persistence of these dynamics in organisational cultures.

Higher education is not immune to these issues (28). Although it is often credited with promoting critical thinking, ethical training, and social progress, in practice, as O'Connor (29) warns, universities also reproduce gender inequalities in their organization, culture, and practices. This contradiction reveals the gap between institutional discourse and everyday realities. GBV in this context is not an abstract concept, as it manifests itself in situations of peer harassment, abuse by figures of power, limited representation of women in leadership positions, and the invisibility of feminist content in curricula (11, 30–33). Furthermore, the difficulties in reporting, accessing protection, and obtaining redress are not simply administrative failures; they reflect mistrust of institutions, impunity for perpetrators, and experiences of revictimization (34). In this scenario, the culture of silence, the normalization of sexism, and the absence of a gender mainstreaming perspective reinforce the urgency of structural and educational transformations (35). Although prevention programs exist, most focus on increasing knowledge about GBV among adolescents and university students, showing limited effectiveness in changing behaviors (36, 37).

The situation in Spanish universities is alarming. Data indicate that 62% of students have experienced or witnessed violence against women, but only 13% initially identified these situations as such (38). Most of the situations of violence identified by students occur between students, not necessarily involving figures of authority. A study with Spanish university students demonstrated a causal link between the failure to recognize violent behavior and the underreporting of incidents (28, 38). Nevertheless, higher institutions can serve as role models in creating safe spaces and setting an example of how to prevent, detect and act in the face of GBV situations.

The urgency of addressing GBV within higher education institutions is now underscored by recent legislative reforms in Spain and Catalonia that have significantly strengthened the legal framework. This development builds on earlier milestones, such as the Ley Orgánica 1/2004, de Medidas de Protección Integral contra la Violencia de Género [Organic Law 1/2004, on comprehensive protection measures against gender-based violence], which established a broad penal, social, and preventive framework (39), and the Ley Orgánica 3/2007, de igualdad efectiva de mujeres y hombres [Organic Law 3/2007, on the effective equality of women and men], which imposed obligations on educational and university institutions in the field of gender equality and training (40). In Catalonia, the Llei 17/2015, d’igualtat efectiva de dones i homes [Law 17/2015, on the effective equality of women and men], also introduced provisions on the integration of gender perspective in higher education (41). Aligned with these foundations and with international standards such as the Council of Europe Convention on Preventing and Combating Violence against Women and Domestic Violence (42), the Istanbul Convention, subsequent reforms have expanded universities’ responsibilities. Among the most relevant are the Llei 17/2020, de modificació de la Llei 5/2008, del dret de les dones a erradicar la violència masclista [Law 17/2020, amending Law 5/2008 on the right of women to eradicate gender-based violence] (43); the Ley 3/2022, de convivencia universitaria [Law 3/2022, on university coexistence] (44); and the Ley Orgánica 10/2022, de garantía integral de la libertad sexual [Organic Law 10/2022, on the comprehensive guarantee of sexual freedom] (45), known as “Solo sí es sí”. These laws expand universities’ obligations by requiring structural prevention and reparation policies, permanent training for academic and administrative staff, swift and non-revictimizing institutional responses, and robust support mechanisms for victims (43–45).

The research legislative framework has also evolved to incorporate explicit measures against gender-based violence and harassment. The Llei 9/2022, de la ciència [Law 9/2022, on science] (46) requires public research institutions to include explicit ethical commitments against sexual harassment, revictimization, and gender-based violence, and to establish mechanisms for victim protection and support. Likewise, the Ley 17/2022, which amends the Ley 14/2011, de la ciencia, la tecnología y la innovación [Law 14/2011, on science, technology and innovation] (47), obliges public research entities to implement and annually monitor protocols addressing sexual harassment as well as harassment based on sex, sexual orientation, gender identity, or sexual characteristics. These reforms also aim to dismantle structural barriers in academia and research, including the glass ceiling, gender pay gaps, and vertical and horizontal segregation (46, 47).

Meanwhile, the *Pacto de Estado contra la Violencia de Género* [State Pact against Gender Violence], first approved in 2017 as a comprehensive framework of coordinated measures, was updated in February 2025 to include new actions against economic, vicarious, digital, and sexual violence, with the involvement of all public administrations and universities (48, 49).

In this social, academic, and legal context, previous studies have addressed the voices of victims in both sports and universities, exploring the manifestations of violence, the reasons for victims’ silence, and the role organizations play in perpetuating this silence (34). Nevertheless, a significant gap remains: the perspective of university teaching staff has not been sufficiently analyzed.

Therefore, the purpose of this study was to explore the perceptions, experiences, and proposals of university teaching staff in relation to gender-based violence in the Physical Activity and Sports Science (PASS) degree. By documenting university teaching staff perspectives, the research aims to contribute to identify gaps between institutional discourse and everyday realities and to develop evidence-based recommendations for effective prevention and intervention in higher education.

Theoretical frameworks have been instrumental in classifying violence into distinct typologies, thereby facilitating its recognition and treatment. Regarding the specific theoretical lenses applied, various studies have examined GBV using models such as the ecological model (17), the theoretical model of network silence (50), and the feminist socio-ecological model (5). Other scholars, meanwhile, have applied feminist theories on violence, feminist sociology, gender studies, and organizational theory (51).

In contrast, this study adopts the conceptual and theoretical framework of the 7P model. This model expands upon established international approaches, such as the UN and EU's 3P framework (Prevention, Protection, Prosecution) and the Council of Europe's Istanbul Convention 4P model (adding Policy) by offering a more comprehensive structure for understanding and addressing GBV. 7P model was specifically created to address gender-based violence, particularly in contexts such as higher education, research, and sports. It was developed within the European UniSAFE project, which aims to build university and scientific environments free from gender-based violence. The model comprises seven dimensions: Policy, Prevalence, Prevention, Protection, Prosecution, Provision of services, and Partnerships (2, 52). Originally applied in the context of sports and GBV (2), the 7P model was subsequently adopted by the UniSAFE project for application in higher education and research organizations (51, 52).

Based on the UNISAFE (52) definitions, in this paper we used the following conceptualization: 1) Policy, defined as a comprehensive set of policies and guidelines to address violence against women. These include developing national action plans, implementing anti-discrimination measures, and promoting the participation of women in decision-making processes; 2) Prevalence, refers to data and data collection aimed at estimating the extent of gender-based violence and at providing information on its different forms. Data can be collected (with intersectional approach) through surveys or administrative processes (e.g., the registration of complaints); 3) Prevention, refers to measures that promote changes in social and cultural behavior, like induction materials for both staff and students; internal and external publicity and training; 4) Protection is about ensuring safety and meeting the needs of (potential) victims and survivors, with the objective to avoid (further) harm being inflicted; 5) Prosecution and disciplinary measures cover legal and disciplinary proceedings against perpetrators, and related investigative measures and judicial proceedings; 6) Provision of services refers to the services offered to support victims, families, bystanders, perpetrators and the community affected by gender-based violence; and 7) Partnerships relate to the involvement of relevant actors at all levels, such as governmental agencies, civil society organisations, trade unions, or staff and student associations.

## 2 Materials and methods

This paper draws on data from the two-year research project “ViQuad: A proposal for educational transformation in gender violence in Physical Activity and Sports Science studies.” developed across three universities offering the Degree in Physical Activity and Sports Science (PASS).

### 2.1 Data collection

A total of three focus groups were held with university teaching staff from the PASS degree program during the course 2024–2025. Participation was voluntary, and written informed consent was obtained from all participants.

A semi-structured focus group guide was developed, informed by the 7P model framework (2). The guide was reviewed by two external experts with experience in GBV and higher education, who provided feedback on the relevance, clarity, and alignment of the questions with the study aims. This framework structured the inquiry to encompass key dimensions of the GBV phenomenon within the institution, guiding questions related to participants’: (1) opinion on gender perspective, (2) opinion on GBV, (3) experiences of GBV, (4) experiences of addressing and responding to GBV, and (5) proposals for responding to GBV. In addition, a critical pretext was introduced (a hypothetical scenario of GBV that could occur in their classroom) to challenge participants to analyze and describe their potential responses. During the focus group, participants were provided with a document containing definitions of the concepts (typologies of GBV), based on the UNISAFE study (28).

The focus groups were conducted between December 2024 and April 2025, and were moderated by two researchers, applying a detailed protocol. Each group lasted approximately 90 min and participants could respond in either Catalan or Spanish.

Given the sensitivity of the topic and participants’ possible hesitation to express their views, it was important to conduct the focus groups using a shared-power, non-coercive approach. Participants were given ample time to express their feelings and thoughts. They were also allowed to leave the focus group at any time, and breaks could be integrated upon request.

### 2.2 Participants

A total of 17 university teaching staff (8 women and 9 men), with university teaching experience ranging from 1 to 43 years (M = 17.68, SD = 10.92), participated in the focus groups.

A purposive sampling strategy was employed to ensure heterogeneity and contextually rich sample, with participant selection guided by criteria aimed at maximising diversity across the following dimensions: academic area (5 in Physical Education, 5 in Sports Performance, 4 in Health, and 3 in Sport and Leisure Management); career stage (3 early-career, 8 mid-career, and 6 senior faculty); academic position (3 associate professors, 5 tenure-track lecturers, and 9 full professors), teaching profile (5 in practical or motor-oriented subjects as well as 12 teaching primarily theoretical courses), and levels of familiarity with gender perspective and GBV. Among the participants, 3 demonstrated high sensitivity to gender issues and were actively involved in their university's equality commission; 7 had previously attended at least one training activity related to gender perspective or GBV, while the remaining 7 reported no prior formal training or substantial awareness in this area.

Recruitment was conducted through university email and personal invitations. Faculty members who expressed interest were subsequently contacted by email by the principal investigator and provided with detailed information about the study objectives and expected outcomes.

Overall, this strategy allowed the inclusion of perspectives shaped by diverse professional trajectories and profiles within university teaching staff in PASS.

### 2.3 Data analysis

Data were analysed using a reflexive thematic analysis, following the principles outlined by Braun, Clarke, and Weate (53). The analytic process combined inductive and deductive phases in an iterative and non-linear manner. This approach allowed themes to emerge from participants’ accounts while situating them within a theoretically informed framework.

The first phase of analysis was predominantly inductive and focused on familiarisation with the data. This involved repeated reading of the transcripts, the writing of initial analytic memos, and the generation of preliminary codes grounded in participants’ own language and experiences. Inductive codes captured recurrent perceptions, uncertainties, and practices related to GBV in PASS contexts. Examples of inductive codes included “*not knowing how to act”, “fear of misinterpreting a situation”, “students’ silence”* and “*uncertainty about reporting procedures”.* These codes functioned as conceptual building blocks, remaining close to the data.

In the subsequent analytic phase, inductively generated codes were examined for patterns and conceptual convergence and were then interpreted deductively using the 7P framework (51, 52) as an interpretative lens. This deductive phase did not involve forcing data into predefined categories; rather, it entailed a reflexive process of theorisation in which emerging codes were related to the dimensions of the 7P model when analytically appropriate. For example, the inductive code “*not knowing how to act”* emerged consistently across focus groups and was subsequently grouped within the deductive theme “Protection”, as it reflected limitations in faculty members’ perceived capacity to ensure students’ safety and to prevent further harm when facing potential situations of gender-based violence. Similarly, inductive codes such as “*uncertainty about sanctions”* and “*focus on perpetrators’ rights”* were interpreted within the “Prosecution” dimension, while “*lack of follow-up with victims”* was aligned with “Provision”. This iterative movement between inductive coding and deductive interpretation ensured that the analysis remained grounded in participants’ accounts while engaging critically with the theoretical architecture of the 7P model.

This analytic strategy enabled a nuanced and context-sensitive exploration of university teaching staff's perceptions and experiences of GBV in PASS degrees, situating individual narratives within broader institutional and structural dynamics.

To enhance analytic rigor and transparency, the coding and theme development process involved multiple stages of researcher collaboration. Two researchers independently conducted the initial inductive coding of all transcripts. This was followed by iterative analytic meetings in which codes, emerging patterns, and thematic boundaries were discussed and refined. Disagreements were addressed through reflexive dialogue aimed at reaching interpretive consensus rather than through quantification or intercoder reliability metrics, in line with the epistemological assumptions of reflexive thematic analysis.

In addition, the analytic process incorporated the involvement of a critical friend (53) with expertise in qualitative methodology and GBV research. The critical friend reviewed the developing themes, questioned analytical assumptions, and challenged interpretive decisions, thereby strengthening reflexivity and reducing the risk of unexamined bias. ATLAS.ti software was used to support the organisation, retrieval, and comparison of codes and themes throughout the analytic process.

### 2.4 Ethical

Ethical considerations such as voluntary participation, the option to skip sensitive questions, and post-session support contributed to the authenticity of the findings by ensuring a respectful and inclusive research environment. The study was approved by the Ethics Committee of the University of Barcelona (CER032511).

## 3 Results

The results of this study are organized according to the 7P model and are illustrated and nuanced through direct quotations from participants’ narratives. To ensure confidentiality and protect participants’ anonymity, all names used in the presentation of the results are pseudonyms.

### 3.1 Prevalence: faculty recognition of severe, verbal, physical, and organizational forms of GBV

In this study, prevalence is approached from a qualitative perspective, drawing on university teaching staff's perceptions and experiences to explore how different forms of GBV are identified, interpreted, and made visible within PASS programs. The results presented below reflect how participants perceive the presence of GBV across academic roles and educational contexts, highlighting both highly visible manifestations and more normalized or less explicitly recognized forms of violence.

Across the focus groups, university teaching staff referred to a variety of forms of GBV, although these were not identified with the same clarity or intensity. Participants’ accounts predominantly focused on the most visible and socially recognized manifestations of GBV, particularly sexual harassment, which emerged as the most recurrent form mentioned in the narratives. As one participant stated, “*There are things! There are situations among students. There has been harassment”* (Julia). Similarly, another faculty member recalled a specific incident:

“In 20 years, I have only had one case of inappropriate touching (I’m not sure how to say it properly). The student in question had a good enough relationship with me to mention it, but she did not want to file a complaint” (Paco).

Other accounts described persistent and intrusive behaviors, such as “a male student who follows female students, also showing interest in where they live or work, and then appearing in places that these women regularly go to. Persistently” (Julia). More severe situations involving violations of privacy were also reported, as illustrated by the following testimony: “There was a situation in the bathrooms where a male student took photographs while a female student from our program was on the other side” (Marga).

Verbal violence was also frequently highlighted in participants’ discourses. This included sexist comments, inappropriate language, and normalized jokes, often framed as behaviors embedded in everyday academic interactions. Examples included remarks such as “*Boys, you don’t need to come, girls, you can come to my office if you want”* (Sofia), as well as the use of sexualized images in teaching materials, described by one participant as

“a case of a lecturer who used precisely those PowerPoint presentations with certain images that were.. With violence that was explicit to me.. Well.. a man on a motorcycle and a girl in a thong with her butt showing and something that had nothing to do with anything..” (Sofia)

In contrast, references to physical violence were less common and were typically described as exceptional or ambiguous situations. One faculty member reported having detected excessive physical proximity during practical classes: “*I am aware that it has happened here. That some male student has been very… very close… too close to a female student. And this has happened in our classrooms; there was even a formal complaint”* (Roberto). However, in other instances, physical contact was framed as more ambiguous and potentially normalized within sport contexts, as reflected in the following comment: “*Violence can also be related to what we are doing, to contact. Contact, because we touch one another, because on the floor we are on top of each other. There are positions that can be… very closely related to sex”* (Paco).

Finally, organizational or institutional violence appeared more indirectly in the participants’ narratives. Rather than being explicitly named, it emerged through references to silences, power asymmetries, lack of institutional mechanisms, and unequal treatment. One participant described a situation of structural inequality as follows:

“A young woman with a man who has been here for many years and who, nevertheless, has the same job category, the same responsibilities, and the same institutional roles… and yet the treatment is absolutely unequal. And… the institution does not have the mechanisms to stop it, generating a clear situation of inequality, lack of protection, and situations that are very difficult to endure” (Felip).

### 3.2 Prevention: GBV-related perceptions, attitudes in physical activity and sport sciences contexts

The concept of *prevention* applied in this article is related to teachers’ perception of the existence of GBV in PASS. If they do not know what GBV is, it will be difficult for them to detect it and act accordingly. In this regard, the teachers who participated in the study reflect on whether they have observed or been aware of any GBV in their university environment. On the one hand, there is a lack of awareness of the problem, as they deny its existence.

“I haven't.. I've never witnessed any explicit violence in all my years at [name of institution], have I? Not in class.. not.. no, no, no, I haven't experienced it. If I had, I would have tried to intervene, but it’s never been necessary”. (Enric)

Furthermore, some university teaching staff acknowledge the potential that, throughout their careers, a lack of training and awareness may have prevented them from identifying witnessed situations as GBV.

“I am sure that at some point this situation must have arisen in 24 years (teaching the subject). Now, I have not had the ability or been well trained to truly discern whether something was happening or not. Therefore, it is likely that it did happen, but I did not have the ability to see it, most likely.” (Felip)

However, all of the university teaching staff who took part in the study were aware that they had witnessed situations of GBV at some point in their academic careers. Some of the most common situations involved the detection of the inappropriate use of women’s bodies in teaching materials. That is, the use of women as objects, sexualized bodies, to attract the attention of students (mostly male), or the omission of women from the subject content.

“PowerPoint presentations with highly sexualized female bodies and without male bodies. Well, something very, very, very.. gender-polarized.” (Julia)

Another form of detected GBV is verbal, occurring within teaching explanations. For instance, one university teaching staff described how to assist in a gymnastic movement by using colloquial language to refer to the buttocks of a female student who was serving as a teaching assistant.

“Some help with an exercise in which I was doing a bridge and was supposed to lift my glute, but instead of explaining it from a technical perspective, they referred to it in a crude, non-technical way” (Sofía).

### 3.3 Policy: formal commitments and the gap between declared frameworks and everyday academic practice

From a *Policy* perspective, the accounts highlight significant gaps in awareness and understanding of the institutional framework designed to address GBV. In the institution analyzed, the *Protocol for the prevention, detection, and response to situations of sexual harassment based on sex, gender identity, and sexual orientation, as well as other sexist behaviors*, is publicly available. In addition, the university has issued an official institutional declaration explicitly stating a policy of zero tolerance toward sexual harassment and harassment based on sex, gender identity, and sexual orientation. Through this declaration, the institution formally commits to: (a) publicly declaring and disseminating its rejection of all forms of sexual harassment and harassment based on sex, gender identity, and sexual orientation; (b) promoting a preventive culture through training and information actions addressed to the entire university community; (c) investigating and, where appropriate, sanctioning conduct that may constitute harassment, in accordance with the established protocol; and (d) accompanying and advising individuals who have experienced harassment.

The institutional framework also includes an *Equality Commission*, which is formally responsible for receiving complaints, monitoring reported cases, and developing actions related to prevention, information, training, and advisory support. From a policy perspective, these structures and documents constitute a coherent formal framework that articulates the institution's declared commitment to addressing GBV.

However, the participants’ accounts reveal a significant gap between the existence of these policies and their translation into shared knowledge and practices among teaching staff. As illustrated in the narratives that follow, many participants were unaware of the content, scope, or activation mechanisms of the protocol, highlighting a disconnect between institutional policy frameworks and the protection processes experienced and enacted at the faculty level.

Several participants reported being unaware of the existence of a prevention and action protocol at the faculty, indicating that policy documents had not been effectively communicated or embedded within everyday academic practice: “*To be honest, I don't know about it, I try to deal with situations as they arise. If something happens, I would go to management and management would tell me what to do” (Paco)*.

Other participants acknowledged knowing that a protocol existed, yet emphasized their lack of familiarity with its content, scope, and operational logic. These narratives illustrate how policies may formally exist but remain disconnected from staff practice when they are not actively disseminated or translated into accessible guidance:

“I don't know the protocol.. I don't know it, and in fact.. when in recent weeks, well, I've gotten started on this, because I think I have to get up to speed.. learn the house rules, eh, and also get them across to the students, one way or another..” (Arantxa)

These accounts point to a distinction repeatedly raised by participants between passive awareness of policy documents and active knowledge required for effective implementation. As Felip noted, the mere existence of a protocol does not guarantee protection if its content is not widely known, understood, and operationalized: *“We must be familiar with the protocols, and this is not just a matter of creating a protocol. It is a matter of teaching this protocol, and perhaps this is still to be done” (Felip).* The participants distinguished between passive awareness and active knowledge, arguing that effective implementation requires widespread circulation and understanding of the document's contents, not merely the fact of its existence.

### 3.4 Protection: barriers to intervention and protocol awareness

The *Protection* dimension becomes particularly visible in participants’ expressions of uncertainty regarding how protocols are activated, what procedural phases they involve, and who is affected at each stage. This lack of procedural clarity undermines staff confidence and limits their ability to act protectively when faced with potential cases of GBV: *“We don't know how the protocol works”(Roser)*.

Participants also emphasized that effective protection requires not only formal procedures but also institutional infrastructures capable of supporting training, information, guidance, and coordinated action to protect. In this regard, teaching staff reflected on the relatively recent establishment of equality structures within the faculty, highlighting both their existence and their limited consolidation over time:

“We haven't even had a protocol for 10 years; it hasn't been that long since we've had this protocol. It hasn't been that long since we've had an equality commission. It hasn't been that long since we've had many of these things, but they do exist. Yes, they do.” (Felip)

Protection in research performing organisations requires clear processes, procedures and infrastructure for reporting occurrences, training and expertise of those responsible for designing and implementing these processes, procedures and infrastructure, and for those acting as contact points. A key component of protection is training, which has the potential to drive profound cultural and behavioral shifts. The findings point to a significant demand for structured GBV education at the university, especially in the PASS degree program. The university teaching staff proposed specific training sessions focused on knowledge-based prevention, including the identification of GBV and familiarization with the university protocol. Discussions also arose regarding the implementation of these measures, particularly whether attendance should be mandatory. These proposed actions are envisioned as essential for raising awareness among both the academic staff and students.

“Training that I think we do, but we should try to make some training optional and other training mandatory. And.. we have to do it! We have to do it! But let’s not just say it now. [..] All teachers who have a permanent position must have a very clear understanding of the common part, whether you want it or not, or I don't know if it’s common or specific.. but we must be trained in it. And we're not. We're not!” (Felip)

These quotes emphasize the lack of awareness of the issue within the faculty. However, they sometimes externalize the need for training to the students, rather than to the teaching staff. This is the case in the following example:

“We lack training and information, right? Because we all know that there is an equality committee, yes, but I insist, and I'm speaking from my own experience, what protocol do they have, what knowledge do the students have, is it explained to them in the first year? I'm in my third year, I don't know what they explain to them, but I'm in my third year.. if nobody tells me this, they explain it to them from the first year.. because I don't attend the first-year session, so I don't know if they get it..” (Roser)

On the other hand, regarding concrete actions aimed at preventing potential victimization, that is, measures adopted on a case-by-case basis, there are few references. Some of the actions mentioned include speaking directly with faculty members against whom a complaint had been filed due to the use of sexualized language or images in their teaching materials:

“A conversation with this lecturer… Complicated, right? Because you are telling him… between the two of you and… even though I am the director, you are telling him… that things were not handled properly and that he needs to change certain aspects… and the situation becomes tense” (Xisco).

### 3.5 Prosecution: faculty responses to GBV in physical activity and sport science context

According to the results of this study, different types of GBV appear in university classrooms teaching the PASS degree. Some of these have been addressed in the previous sections, although the measures implemented to combat such GBV have not been mentioned. This section analyzes the measures implemented in response to GBV (prosecution of the 7P's model). It should be noted that this is analyzed from the perspective of the university teaching staff, as it could be that the faculty has applied some type of sanction and the teaching staff is not aware of it.

There are several interventions that describe situations in which, when faced with verbal abuse, the students themselves file a formal complaint through the university equality committee. In this regard, Júlia comments:

“Clearly sexist comments made in class in front of everyone, and no one reacted at all, right? You say how is it possible that no one said anything.. you say it now and.. someone (referring to a female student) is sure to jump in, well.. hopefully that’s the case, but not so many years ago then.” (Júlia)

Furthermore, this last quote also shows the evolution of awareness in classrooms. It reflects that a few years ago, students themselves would not have complained about the existence of this violence. However, today, when faced with verbal violence (whether through PowerPoint presentations, verbally, or in writing in their documents), students themselves are aware of it and complain to the institution.

On the other hand, sometimes it is the students themselves who make comments that could be classified as GBV. Therefore, it is the university teaching staff who is responsible for responding to this type of violence. University teaching staff admit that they do not know how to react, and their responses vary greatly. Javier's response stands out, in which he explains that he tries to do so with humor so that the students who have shown their displeasure do not trigger a conflict:

“Right in class, I asked, we asked, ‘What does entrepreneurship mean to you?’ And..and one of them went..Well, we did it interactively, we put it on the screen: balls, right? I mean..you have to have balls to be an entrepreneur, and I immediately saw that..there were two or three girls in the front row who didn't take it too well, right? And a mini debate ensued..because in the end I laughed too..and I said, well, you'd be a testicular entrepreneur, right? I mean, you..we start businesses because of..because of testicles..so, we joked around and..well, we got a little tough, right?” (Javier)

### 3.6 Provision: mapping support gaps/controversy and decision paralysis in GBV response

The next section of the results addresses the provision. As a reminder, this provision refers to the services offered to victims of GBV. In this section, we address the lack of follow-up and support for victims affected by GBV known to the university teaching staff participating in the study. Some comments made by teachers when they have been aware of violence specify that they have not been able to help. In this regard, Paco refers to a case he experienced firsthand, in which a student revealed to him that she had been a victim of GBV by a classmate during his class:

“In 20 years, I've only had one case of inappropriate touching..The girl in question had a good enough relationship with me to mention it, but she didn't want to report it. And that’s a problem, because I'm aware of this, but if she doesn't want to report it..we're always in..Naturally, after this, I'm obviously more aware. But if they don't report it at the institutional or judicial level, even though I insisted” (Paco).

In this case, the teacher encouraged the victim to report the incident to the institution. However, the student did not want to report it. The university teaching staff in this case concluded that he could not take any further action with the student concerned. From another point of view, the teacher stated that, since that incident, he had been more attentive in his class to prevent further incidents from occurring. It should be noted that this same teacher is not familiar with the protocol or the reporting mechanism. In this regard, the statement that he would “be more attentive” in his class raises questions about what indicators he would be looking for to prevent GBV and what type of GBV he expects to observe.

Another situation worth highlighting is the lack of knowledge among university teaching staff about the tools available to them, as well as the lack of information among those who lead the institution to support victims, when there is still no protocol for GBV prevention and action. In this regard, one student was the victim of GBV by a group of students. When the institution became aware of the situation, they acknowledged that they lacked experience and that it was difficult to act due to a lack of resources and measures. Similarly, they acknowledge that they do not know what measures to apply, as they do not want to apply measures that are too harsh, but at the same time too lenient:

When it came to me, and..Well, we had to deal with the case,..um..which wasn't going to be easy, because..we don't have much experience..and that was one of the reasons why we ended up generating..among other things, right? Because..there was no help from equality..we did the consultations openly..Well, to say how to proceed, right? Because, you know..you don't want to go overboard, but you don't want to fall short either, right? (Xisco)

Once again, however, they focused on the aggressor. They ensured that the measures applied to him would not cause him excessive discomfort. However, they did not mention anything about the victim or her support.

In this sense, in some cases, the provision is nullified by the concern that the aggressor will retain his rights, especially if he is a member of the teaching staff. In this regard, Jofre's contribution is noteworthy, referring to one case of GBV committed by a teacher against several female students:

“You must always respect the rights of anyone who feels abused, assaulted, or violated..but also from the point of view, especially if it is a..member..of the teaching staff, whether permanent or not..you have to be very careful when..taking any..any kind of decision on the matter, which we are all aware has happened this first semester, has not been very scrupulous with the procedure..because people also have labor rights, however abusive..” (Jofre)

### 3.7 Partnership: limited and unknown collaboration with internal and external actors

In the institutional context analyzed, formal collaboration mechanisms do exist to provide psychological support for victims and to implement educational and restorative processes with perpetrators. However, these collaborative processes were not identified or mentioned by the university teaching staff participating in the focus groups, indicating a limited awareness of such partnerships.

Overall, the (no)results show a clear gap between existing institutional-level partnerships and faculty members’ knowledge of these collaborative actions, with partnership remaining largely invisible in the everyday discourses and practices of university teaching staff.

## 4 Discussion

This study contributes to the field of sports politics, policy, and law by examining how GBV is understood, addressed, and governed within PASS university programs. Using the 7P model as an analytical framework, the findings reveal that GBV in this context is not merely an interpersonal issue but a structural governance problem, shaped by policy gaps, institutional silences, and weak implementation mechanisms. The results highlight a persistent disconnection between formal regulatory frameworks and the everyday practices through which sport-related academic environments are organized and managed.

In our study, faculty staff reported being able to identify and witness various forms of GBV present within the university environment throughout their careers, including sexual harassment and verbal, physical, and organizational forms of GBV. Previous research, such as the study by Humbert et al. (2024), indicates that certain typologies, like sexual and psychological harassment, are among the most reported in surveys in the university setting. It is noteworthy that while GBV was initially discussed by participants as a distant phenomenon not prevalent in academia, every contributor to this study was ultimately able to describe at least one case they had witnessed or learned about during their professional trajectories at PASS University.

Prevention emerges as a central but underdeveloped pillar. The results demonstrate that university training initiatives, widely recognized as key instruments for challenging gender stereotypes in sport (54), remain fragmented and insufficiently embedded within PASS programs. This aligns with broader research indicating that higher education institutions often lack systematic, well-resourced preventive strategies capable of reducing GBV prevalence or ensuring early intervention (51).

In sport-related academic contexts, prevention is not merely an educational task but a governance responsibility. The limited training received by PASS faculty reflects a broader policy failure to integrate gender equality and GBV prevention into the core regulatory and curricular structures of sport education. Without mandatory, context-specific training that challenges entrenched gender norms and power relations in sport, universities risk reproducing the inequalities they are formally committed to eradicating. Effective prevention, therefore, requires institutional policies that move beyond declarative commitments and translate gender equality objectives into enforceable educational standards and accountability mechanisms (5, 6).

The protection dimension reveals how weak dissemination of institutional policies and protocols undermines accountability within the university. Although formal protocols exist, teaching staff frequently reported uncertainty regarding their content, activation procedures, and the actors responsible for their implementation. In the present study, faculty members also described situations in which victims were reluctant to report incidents, reflecting not only fear of reprisal but also a profound lack of trust in existing institutional mechanisms. This lack of operational clarity generates insecurity among potential interveners and reinforces a culture of institutional silence, a pattern well documented in higher education governance research (34, 38, 55).

From a policy and regulatory standpoint, protection mechanisms fail when institutional rules are not accompanied by clear communication, training, and oversight. The findings suggest that the mere existence of protocols is insufficient; effective protection requires that policies be actively internalized by staff and embedded within everyday governance practices. In the absence of such integration, protection becomes uneven, discretionary, and dependent on individual initiative rather than institutional obligation, undermining universities’ duty of care toward students and staff (56).

The prosecution dimension exposes tensions between due process, institutional reputation, and victim-centred justice. Faculty members’ hesitation to intervene or facilitate reporting reflects not only role confusion but also a broader lack of institutional support for those tasked with enforcing policy at the ground level (57). This situation reveals a governance paradox: while universities are formally responsible for ensuring safe environments, they often fail to equip staff with the authority, protection, and training required to enact disciplinary processes effectively (58).

The literature indicates that policy instruments such as bystander intervention programs can strengthen institutional capacity by clarifying roles and fostering collective responsibility (59). In sport-related academic contexts, where power hierarchies and informal networks are particularly pronounced, such interventions are essential to counteract normalization of abuse and reluctance to challenge peers or superiors.

In the present study, the provision of services constitutes a critical weakness in the institutional response to GBV. The findings show that organizational responses tend to prioritize the procedural rights and reputational protection of alleged perpetrators, while victim support remains marginal or implicit. This pattern can be understood through gendered organizations theory (60, 61), which highlights how institutional arrangements privilege stability, hierarchy, and collegial protection, values historically embedded in sport governance structures (11).

In PASS programs, these gendered governance regimes shape how risk and responsibility are allocated, often reinforcing silence and discouraging transformative action. Effective provision requires the development of specialized, visible, and accessible support services that address not only emotional harm but also the academic and professional consequences of GBV (62, 63).

Finally, the findings point to significant shortcomings in partnership and policy coherence. Although institutional protocols are formally reviewed in collaboration with external departments and, in some cases, involve referrals to specialized foundations, these partnerships remain largely invisible to teaching staff. This lack of transparency limits their effectiveness and reinforces fragmented governance. The absence of visible partnerships reflects a broader policy challenge: the failure to align institutional policies with implementation networks capable of addressing GBV across the multiple spaces in which sport education occurs (34, 57, 59).

Strengthening the policy dimension therefore requires not only clearer regulations but also mechanisms for monitoring, accountability, and stakeholder engagement. As highlighted by Hagerlid et al. (65), effective institutional governance depends on addressing both cognitive gaps, through clear definitions and education, and trust deficits, through transparent procedures and credible enforcement. In the context of PASS programs, this implies rethinking GBV not as an isolated risk but as a central issue of sport governance, institutional responsibility, and policy effectiveness.

## 5 Conclusion

This study contributes to the existing literature by providing qualitative evidence from faculty members in PASS degree, an underexplored context in research on GBV in higher education. The results indicate that faculty members are able to identify the most serious cases of sexual and physical violence and are aware of the existence of structural violence. However, the results reveal a critical gap between recognition and action: most participants reported uncertainty about how to intervene in GBV situations occurring in classrooms or among students and demonstrated limited knowledge of the procedures required to activate institutional protocols. This gap underscores the need for targeted institutional measures that move beyond awareness and enable concrete, supported action by teaching staff.

The consistency between our results and prior literature (1, 13, 28, 64, 67), which identifies athletic contexts as high-risk environments, reinforces the urgency of integrating GBV prevention into the core of sport and physical activity education. The findings show that universities and sport-related institutions continue to reproduce gendered organizational dynamics that hinder effective responses to GBV (66). Faculty hesitation to intervene, often justified by concerns about reputational damage, procedural ambiguity, or institutional conflict (3), reflects broader gendered organizational processes (60, 61) in which protecting colleagues and institutional stability is prioritized over victim-centred responses. The convergence between our findings and existing literature (1) confirms that these challenges are not isolated but systemic across higher education and sport settings.

The application of the 7P model provided a structured framework to translate these findings into actionable institutional priorities. The lack of training in gender perspective and GBV among teaching staff highlights the need for mandatory, discipline-specific education. Universities should implement compulsory training for PASS faculty and students that includes: (a) recognition of subtle and normalized forms of GBV; (b) practical bystander intervention strategies tailored to sport and physical activity contexts; and (c) explicit discussion of power relations and gender norms in sport. Such training should be embedded within PASS curricula and staff development plans rather than offered as optional or one-off initiatives.

The university teaching staff participating in the study were receptive to the topic, highlighting the need for spaces for training and collective reflection that would enable them to identify, understand, and act on GBV from their educational role. The experience in the discussion groups revealed the pedagogical value of participatory and dialogical methodologies, in which the voice of the university teaching staff becomes a central element for institutional and cultural change.

The findings demonstrate that the existence of protocols alone is insufficient. Universities should ensure systematic dissemination of protocols through clear, accessible formats and regular training sessions that clarify when and how staff are expected to act. Such training must transcend narrow legalistic or bureaucratic interpretations of harassment, building instead on practical knowledge and the lived experiences of victims to foster genuine competence in identification, intervention, and the creation of zero-tolerance environments. Establishing designated contact persons within departments and integrating protocol briefings into departmental meetings are concrete steps that could reduce uncertainty and institutional silence.

Universities should develop transparent, victim-centred disciplinary pathways that balance due process with accountability, and explicitly define the role of teaching staff in reporting and follow-up processes. Evidence from this study supports the implementation of bystander and disclosure-response training to strengthen staff confidence and reduce hesitation when responding to student disclosures (57). Universities should establish specialized, multidisciplinary support services that provide academic, psychological, and professional assistance from the earliest stages of disclosure, regardless of whether a formal complaint is pursued. Such services should be clearly communicated to staff and students and integrated into institutional equality and well-being structures.

This study is not without its limitations, which in turn delineate productive avenues for future research. First, the voluntary and self-selected nature of the sample may have introduced self-selection bias, as faculty members who chose to participate may have been more sensitive to or interested in issues related to gender perspective and GBV. This may have influenced the findings by accentuating reflective, critical, and training-oriented discourses, as well as the emphasis placed on institutional gaps and perceived lack of preparedness. Consequently, the perspectives presented may not fully capture those of faculty members who are less engaged with or more resistant to addressing GBV. However, it is important to note that the study does not aim for statistical representativeness but for in-depth, contextually grounded understanding of how GBV is perceived and negotiated within Physical Activity and Sport Sciences programs.

Second, the absence of student voices, particularly those from historically marginalised groups. This omission precludes a wider analysis of how GBV interacts with other structural inequalities like sexual orientation, race, disability, and social class to create differentiated experiences of vulnerability, access to justice, and protection. Future research should therefore prioritise incorporating these perspectives and adopting an explicit intersectional approach to achieve a more comprehensive understanding of GBV in PASS degree.

In conclusion, this research affirms the persistent and pervasive nature of GBV in university settings. It demonstrates that eradicating GBV from the fields of sport and physical activity education demands a commitment that extends far beyond the drafting of institutional protocols. It requires a cultural and institutional transformation grounded in the coordinated understanding and implementation of the seven dimensions of the 7P model. Strengthening and articulating these dimensions enables a comprehensive and effective institutional response to gender-based violence in university and sport contexts. By equipping teaching staff with concrete tools, clear responsibilities, and institutional support, universities can move toward safer, more equitable, and accountable academic and sporting environments.

## Data Availability

The original contributions presented in the study are included in the article/Supplementary Material, further inquiries can be directed to the corresponding author.
